# COVID-19 infection and tobacco smoking

**DOI:** 10.17179/excli2021-4242

**Published:** 2021-10-07

**Authors:** Tomoyuki Kawada

**Affiliations:** 1 Department of Hygiene and Public Health, Nippon Medical School 1-1-5 Sendagi, Bunkyo-Ku, Tokyo 113-8602, Japan

## ⁯⁯⁯

***Dear Editor,***

Several reports have concluded that smoking might be partly associated with a decreased risk of coronavirus disease-2019 (COVID-19) infection, and that the "nicotinic hypothesis" can be applied to the preventive and therapeutic strategies for COVID-19 (Tindle et al., 2020[7]; Changeux et al., 2020[1]). Here, I present recent information regarding the association between smoking status, COVID-19 infection, and subsequent clinical outcomes.

Saurabh et al. (2021[5]) conducted a case-control study to evaluate the clinical outcomes of COVID-19 patients who were current smokers. The odds ratios (ORs) (95 % confidence intervals [CIs]) of developing symptomatic COVID-19 infection for those who were current tobacco smokers and/or used smokeless tobacco were 0.46 (0.26-0.78) and 0.81 (0.55-1.19), respectively. Although the authors speculated that the cross-protection from symptomatic COVID-19 infection is afforded by frequent upper respiratory tract infections that are common among tobacco smokers, they could not present scientific evidence of the cross-protection provided by smoking to decrease the risk of symptomatic COVID-19 infection.

Farsalinos et al. (2020[3]) conducted a meta-analysis to examine the prevalence of current smoking among hospitalized patients with COVID-19 in China. The pooled prevalence (95 % CI) of current smoking was 6.5 % (4.9-8.2 %), which was about one-fourth of the prevalence of smoking in the general population. They speculated that the immunomodulatory effects of nicotine might contribute to the reduction in the risk of developing COVID-19 infection. The same authors conducted a re-analysis to calculate the prevalence OR (POR) of smoking among hospitalized COVID-19 patients and also examined the association of smoking with disease severity and mortality of COVID-19 infection via a meta-analysis (Farsalinos et al., 2021[2]). The adjusted POR (95 % CI) was 0.24 (0.19-0.30), and the pooled ORs (95 % CIs) of disease severity and mortality for smoking were 1.40 (0.98-1.98) and 1.86 (0.88-3.94), respectively. Although there are long-term health risks of smoking, the authors suggested that pharmaceutical nicotine or other nicotinic cholinergic agonists might help prevent the development of symptomatic COVID-19 infection.

Regarding disease progression in hospitalized patients with COVID-19 infection, Farsalinos et al. (2020[4]) conducted a meta-analysis. The pooled OR (95 % CI) of adverse outcomes for current smokers compared with that for non-smokers and former smokers were 1.53 (1.06-2.20) and 0.42 (0.27-0.74), respectively. Smoking was related to disease progression in hospitalized patients with COVID-19, although the reason for poor clinical outcomes in former smokers could not be determined. I suppose that there may be health problems that could appear even after smoking cessation. In contrast to a primary preventive effect of smoking on developing COVID-19 infection, smoking was associated with adverse effects and disease progression in COVID-19 patients.

Another meta-analysis presented the risk of poor clinical outcomes in COVID-19 patients who were former smokers. Simons et al. (2021[6]) conducted a meta-analysis to evaluate the association of smoking status with infection, hospitalization, disease severity, and mortality from severe acute respiratory syndrome coronavirus 2 (SARS-CoV-2)/COVID-19 disease. The pooled relative risk (RR) (95 % credible interval [CrI]) of SARS-CoV-2 infection for current smokers compared with that for never-smokers was 0.74 (0.58-0.93). In addition, the pooled RRs (95 % CrIs) of SARS-CoV-2 hospitalization, greater disease severity, and mortality for former smokers compared with those for never-smokers were 1.20 (1.03-1.44), 1.52 (1.13-2.07), and 1.39 (1.09-1.87), respectively. However, the risk of a poor prognosis in COVID-19 patients who were current smokers could not be identified. The comorbidities of current and former smokers should be precisely evaluated to elucidate the differences in prognosis.

Regarding the “nicotine hypothesis”, Usman et al. (2020[8]) reviewed the papers reporting that nicotine might have a protective effect against COVID-19 infection. There existed some biases in epidemiological studies supporting this smoker's paradox. Nicotine has an anti-inflammatory effect and its use results in a blunted immune response, which reduces the severity of the cytokine storm. Moreover, increased nitric oxide in the respiratory tract due to nicotine use might inhibit the replication of SARS-CoV-2 and its entry into the cells. In any case, clinical trials are needed to verify the efficacy and safety of administering nicotine medication to prevent COVID-19 infection.

## Conflict of interest

The author declares no conflict of interest.
